# Technology-Based Psychological Interventions for Young Adults With Early Psychosis and Cannabis Use Disorder: Qualitative Study of Patient and Clinician Perspectives

**DOI:** 10.2196/26562

**Published:** 2021-04-05

**Authors:** Ovidiu Tatar, Amal Abdel-Baki, Christophe Tra, Violaine Mongeau-Pérusse, Nelson Arruda, Navdeep Kaur, Vivianne Landry, Stephanie Coronado-Montoya, Didier Jutras-Aswad

**Affiliations:** 1 Research Center Centre Hospitalier de l’Université de Montréal (CRCHUM) Montreal, QC Canada; 2 Department of Psychiatry and Addiction Faculty of Medicine Université de Montréal Montreal, QC Canada; 3 Lady Davis Institute for Medical Research Jewish General Hospital Montreal, QC Canada; 4 CIUSSS du Centre-Sud-de-l'Île-de Montréal Direction régionale de santé publique Montreal, QC Canada; 5 Faculty of Medicine Université de Montréal Montreal, QC Canada; 6 Institut universitaire sur les dépendances Montreal, QC Canada

**Keywords:** psychology, intervention, cannabis misuse, cannabis use disorder, young adult, clinician, psychosis, schizophrenia, dual diagnosis, qualitative, acceptability, technology-based, telemedicine, mHealth, digital health, eHealth, application, smartphone, mobile phone

## Abstract

**Background:**

The persistence of cannabis use disorder (CUD) in young adults with first-episode psychosis (FEP) is associated with poor clinical and functional outcomes. Face-to-face psychological interventions are effective in treating CUD. However, their use in early intervention services (EISs) for psychosis is inconsistent because of barriers, including high workload and heterogeneity in training of clinicians and lack of motivation for treatment among patients. Tailoring new technology-based psychological interventions (TBPIs) to overcome these barriers is necessary to ensure their optimal acceptability.

**Objective:**

The aim of this study is twofold: to explore psychological intervention practices and intervention targets that are relevant for treating CUD in individuals with early psychosis and to explore factors related to the development and implementation of a technology-assisted psychological intervention.

**Methods:**

A total of 10 patients undergoing treatment for FEP and CUD in EISs participated in a focus group in June 2019. Semistructured individual interviews were conducted with 10 clinicians working in first-episode clinics in the province of Québec, Canada. A hybrid inductive-deductive approach was used to analyze data. For the deductive analysis, we used categories of promoting strategies found in the literature shown to increase adherence to web-based interventions for substance use (ie, tailoring, reminders, delivery strategies, social support, and incentives). For the inductive analysis, we identified new themes through an iterative process of reviewing the data multiple times by two independent reviewers.

**Results:**

Data were synthesized into five categories of factors that emerged from data collection, and a narrative synthesis of commonalities and differences between patient and clinician perspectives was produced. The categories included attitudes and beliefs related to psychological interventions (eg, behavioral stage of change), strategies for psychological interventions (eg, motivational interviewing, cognitive behavioral therapy, psychoeducation, stress management), incentives (eg, contingency management), general interest in TBPIs (eg, facilitators and barriers of TBPIs), and tailoring of TBPIs (eg, application needs and preferences, outcome measures of interest for clinicians).

**Conclusions:**

This study provides a comprehensive portrait of the multifaceted needs and preferences of patients and clinicians related to TBPIs. Our results can inform the development of smartphone- or web-based psychological interventions for CUD in young adults with early psychosis.

## Introduction

### Background

Cannabis is one of the most commonly used substances worldwide, with an estimated annual use of 2.5% in the global population [1]. In Canada, cannabis was legalized for recreational use in 2018, and a population survey in the province of Québec one year after legalization showed that cannabis use in the previous 12 months was highest among individuals aged 18 to 24 years (38%), approximately 50% of whom had moderate or high risk of developing problematic cannabis use, especially among those reporting psychological distress [2]. Mental health is a key modulator of the risk of harms associated with this substance, as illustrated by the high prevalence of cannabis use disorder (CUD) in young adults with first-episode psychosis (FEP; 42%-53%) [3,4]. In this population, persistent cannabis misuse is associated with a longer duration of untreated psychosis [4], increased severity of psychotic and affective symptoms [3,5,6], higher rates of psychotic relapses and hospitalizations [3,7-9], poor psychosocial functioning [3,5,6], lower medication adherence [8,10,11], and lower housing stability [3].

Face-to-face psychological interventions that employ cognitive behavioral therapy (CBT) and motivational enhancement therapy (MET) are effective in decreasing the frequency of cannabis use and severity of dependence in individuals with CUD [12]. A survey of Canadian early intervention services (EISs) for psychosis showed that only 12% offered formal services to address cannabis misuse. This study also highlighted many barriers to adequate implementation of face-to-face psychological interventions for cannabis addiction, including low motivation of patients to change their cannabis use, heterogeneity in staff training and treatment goals (eg, harm reduction [HR] vs lower cannabis consumption), and limited access to treatment because of transportation barriers and restricted clinic hours [13].

Using internet-based psychological interventions can circumvent some of these barriers and pave the way toward more homogenous cannabis misuse–focused programs that can be accessed via EISs. In their systematic review and meta-analysis, which included 2963 participants *without psychosis*, Olmos et al [14] found that internet-based psychological interventions comprising CBT and motivational interviewing (MI) were effective in decreasing the frequency of cannabis consumption. To improve their efficacy in treating CUD, the design of web-based interventions (eg, type of psychotherapeutic techniques used, number of modules, intervention length) must be optimized and solutions to address low engagement of users (eg, high attrition, small number of logins) must be implemented [15-18]. A systematic review of technology-based psychological interventions (TBPIs) to address problematic cannabis use in people *with psychosis* found that none of the included studies used internet-based psychological interventions for these patients and that CBT was not incorporated in any of the TBPIs [19], despite existing evidence at the time the review was conducted that CBT was effective in decreasing the quantity of cannabis used in this population [20]. Nevertheless, the review highlighted that using qualitative methodologies to elicit patient and clinician treatment preferences could help improve the content of psychoeducational videos, engagement in the interventions, and cannabis use–related outcomes [19].

The available body of evidence underlines the importance of promptly addressing cannabis misuse in people with psychosis, the potential of TBPIs (eg, internet-based technologies, text messages) in decreasing cannabis use in this population, and the paucity of studies investigating barriers and facilitators of TBPIs for decreasing cannabis consumption in EISs for psychosis.

### Objectives

The objectives of this study are to explore the perspectives of clinicians and patients on (1) psychological intervention practices and intervention targets that are relevant for treating CUD in individuals with early psychosis and (2) factors related to the development and implementation of a TBPI for CUD.

## Methods

### Study Design

We used a qualitative study design and qualitative description methodology to collect, analyze, synthesize, and interpret data [21,22]. This study was approved by the Research Ethical Committee of the Centre hospitalier de l'Université de Montréal (University of Montreal Health Centre, CHUM; 19.067).

### Participants and Study Setting

We explored the perspectives of 2 distinct groups of participants: 10 patients and 10 clinicians. For patients, we used purposeful homogenous sampling [23] and used the following eligibility criteria: (1) age range of 18 to 35 years; (2) diagnosed with psychotic disorders and CUD based on the Diagnostic and Statistical Manual of Mental Disorders-V criteria; (3) willingness to access interventions to stop or reduce cannabis use; (4) receiving treatment at CHUM’s EIS for psychosis (the jeune adultes souffrant de psychose [JAP] clinic); (5) able to consent to participate in the study; and (6) fluent in French. Clinicians working in first-episode clinics in Québec were eligible to participate. The JAP clinic, located in downtown Montreal with a catchment area of approximately 230,000 inhabitants, offers a range of biopsychosocial interventions for psychosis, including pharmacotherapy, family interventions, psychoeducation, CBT, and interventions for comorbid substance use disorders (SUDs), both in individual and group formats. The clinic follows the provincial guidelines for Québec EIS for psychosis [24]. Currently, TBPIs are not offered as part of EISs for psychosis in Québec.

To recruit clinicians, we used a hybrid purposeful sampling strategy to account for *similarities* (ie, all clinicians were active in clinics in Québec offering EISs for psychosis, including the JAP clinic) and *variations* pertaining to specialty (eg, physicians, nurses, social workers) and location of the clinic (ie, metropolitan or urban), as these factors result in different challenges in offering services [23].

### Study Procedure and Data Collection

Clinicians at the JAP clinic identified eligible patients and made the first approach to seek their interest in participating in the study. Then, a research assistant (VL), who was not involved in clinical care, contacted interested patients, explained the study, obtained their written consent, and invited them to the focus group (June 2019). For patients, we used the focus group method that facilitates the collection of rich data by enabling a dynamic exchange of opinions between participants with similar lived experiences. The research project was presented to clinicians working at the JAP clinic, and the research assistant scheduled face-to-face interviews and provided additional information about the study. We collected data from clinicians using individual interviews because of the heterogeneity of these participants in terms of professional background (eg, physicians, social workers), experience, and responsibilities in EISs for psychosis. The clinicians signed a consent form on the day of the interview. Selected clinicians from the Québec Programs Association for First Psychotic Episodes were invited by email, and the research assistant contacted those interested in participating over phone and provided consent electronically.

Patients and clinicians completed an anonymous sociodemographic questionnaire at the beginning of the focus group or interviews. A moderator (NA) with a background in anthropology and extensive experience in qualitative research in the field of mental health and addiction facilitated the focus group using a semistructured interview guide with open-ended questions developed a priori by the authors based on their review of the literature and consultations with experts (Multimedia Appendix 1). To minimize possible bias during data collection, we invited a moderator affiliated with another institution. The moderator was not involved in participant recruitment and did not know the participants. The focus group was held in French and audio recorded. Participants’ opinions were summarized by the research assistant, presented back to participants by the moderator, and validated by the participants at the end of the focus group. We assessed participants’ involvement in the focus group discussions and concluded that we had sufficient data to answer our research question.

From July to September 2019, semistructured individual interviews were conducted with clinicians by the focus group moderator and were audio recorded. Interviews were conducted in person with clinicians at the JAP clinic and via Skype videoconferencing with clinicians in other locations. The recruitment of clinicians was discontinued once the amount of new information collected during the interviews decreased significantly, indicating data saturation. An interview guide was developed a priori by the authors (Multimedia Appendix 2).

Data were collected in French, audio recorded, and transcribed by a specialized transcription firm, and quotations from transcripts used to substantiate results were translated into English by a professional translator. The transcripts were reviewed for accuracy by the research team before data analysis. Patients were compensated Can $50 (US $38) in the form of supermarket gift cards and clinicians were compensated Can $100 (US $76) for their time and participation in the study.

### Data Analysis

We used a hybrid deductive-inductive approach to analyze data [25,26]. For the deductive analysis, we developed an analytic framework that combines the main topics of the interview guides with the categories of promoting strategies (ie, tailoring, reminders, delivery strategies, social support, and incentives), which were identified by Milward et al [27] in their systematic review as the most important strategies for increasing participation in web-based interventions for substance use. For the inductive analysis, we generated new themes and subthemes through an iterative process that involved multiple reviews of qualitative data. We used a sequential approach for data analysis: in phase 1, we organized the patient focus group data into themes and subthemes. In phase 2, the results of the first phase were used to inform the deductive analysis of the semistructured individual interviews with clinicians and new themes were created inductively. In phase 3, we integrated the results of the first 2 phases into a common thematic structure and synthesized and interpreted data by comparing the opinions of patients and clinicians. Trustworthiness, rigor, and verification of the data were established through intercoder agreement. Researchers with different backgrounds (family medicine [OT], psychiatry [CT], and nursing [VM]) and a patient partner (CA) independently coded the raw data, and discrepancies were resolved through discussions between coders. OT was involved in coding all the data with additional contributions from CT, VM, and CA. The results of each phase were validated by senior researchers (DJ, AA, and NK). The use of NVivo software (V.11; QSR International) facilitated data management and analysis. Relevant patient (P) and clinician (C) quotes are provided.

## Results

### Overview

**Figure 1:**
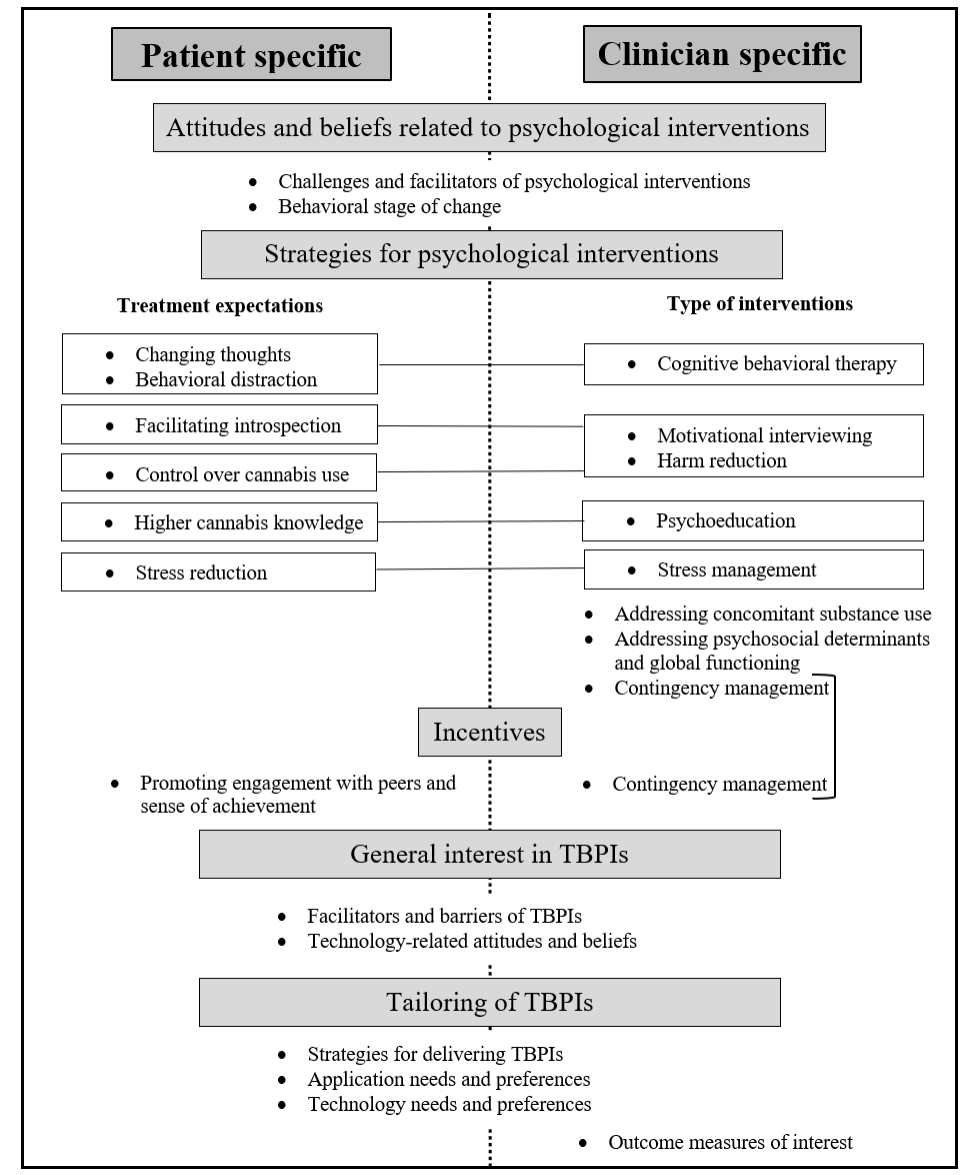
Integration of patient and clinician perspectives related to technology-based psychological interventions. TBPIs: technology-based psychological interventions.

A total of 11 patients and 10 clinicians were invited to participate in the study; all, except one patient, agreed to participate and signed the consent form. The duration of the focus group was 75 minutes, and the mean duration of the individual interviews was 37.2 (SD 6.8) minutes (range 25-51 min). The participants’ characteristics are presented in Tables 1 and 2. Thematic analysis yielded five main themes (Figure 1).

**Table 1:** Sociodemographic characteristics of patients (n=10).

Category		Value, n (%)
**Age group (years)**		
	20-29	8 (80)
	30-39	2 (20)
**Born in Canada**		
	Yes	8 (80)
	No	2 (20)
**Years lived in Canada**		
	Less than 10	0 (0)
	More than 10	10 (100)
**Biological sex**		
	Male	7 (70)
	Female	3 (30)
**Gender**		
	Man	6 (60)
	Woman	2 (20)
	Transgender man	2 (20)
**Race or ethnicity**		
	White	7 (70)
	Asian	1 (10)
	Metis	1 (10)
	Black	1 (10)
**Marital status**		
	Single	9 (90)
	Stable relationship	1 (10)
**Educational attainment**		
	Primary school	1 (10)
	Secondary school—not graduated	3 (30)
	Secondary school diploma	4 (40)
	Professional school	1 (10)
	University undergraduate	1 (10)
**Employment status**		
	Full time	2 (20)
	Part time	1 (10)
	Student	3 (30)
	Sick leave or invalidity	2 (20)
	Other	2 (20)
**Income per year, Can $ (US $)**		
	<10,000 (<7692)	2 (20)
	10,000-20,000 (7692-15385)	4 (40)
	Do not know	3 (30)
	Prefer not to answer	1 (10)

**Table 2:** Sociodemographic and professional characteristics of clinicians (n=10).

Category		Value, n (%)
**Clinic location**		
	Metropolitan	6 (60)
	Urban	4 (40)
**Age group (years)**		
	20-29	1 (10)
	30-39	4 (40)
	40-49	5 (50)
**Gender**		
	Man	2 (20)
	Woman	8 (80)
**Professional occupation**		
	Nurse	2 (20)
	Case manager (sexologist)	1 (10)
	Case manager (occupational therapist)	3 (30)
	Case manager (social worker)	1 (10)
	Psychiatrist	3 (30)
**Clinical experience (years)**		
	4-5	2 (20)
	6-10	4 (40)
	11-20	3 (30)
	>20	1 (10)
**Clinical experience in treating psychosis and CUD^a^ (years)**		
	<5	2 (20)
	6-10	5 (50)
	11-20	3 (30)
**New patients with psychosis and CUD per month**		
	1 to 5	9 (90)
	6 to 10	1 (10)
**Total patients with psychosis and CUD per month**		
	1-5	1 (10)
	6-10	2 (20)
	11-20	6 (60)
	21-30	1 (10)

^a^CUD: cannabis use disorder.

### Attitudes and Beliefs Related to Psychological Interventions

#### Challenges and Facilitators of Psychological Interventions

##### Cognitive Functioning

Clinicians considered cognitive impairment as an important determinant of the efficacy of psychological interventions. In patients’ opinion, new skills acquired during psychological interventions facilitate long-lasting effects of the intervention on cannabis consumption. Meanwhile, clinicians were concerned about patients’ ability to use new acquired skills:

Patients often don’t take in the information and then they can’t correctly identify the factors of relapse, you know, and among other things because of the damn cognitive issues....

C7

Although patients felt that psychological interventions provided a simulation instead of an accurate reflection of real life:

Why do I think psychotherapy doesn’t really work the way it should? Because it doesn’t reproduce the right environment like...what the person is naturally confronted with, like in their daily life. Like, it tries to create a simulation.

P7

clinicians surmised that these impressions may be attributed to patients’ limited ability to process information. Clinicians acknowledged that poor and fluctuating cognitive functioning represented an important barrier to psychological interventions by limiting patients’ capacity to engage in introspection, understand information, plan activities, and attend scheduled therapy sessions. In addition, reduced verbal communication abilities in patients represented a challenge for psychological interventions:

Me, I think that the patients have a hard time expressing their emotions. They struggle with putting their thoughts into words. They still have problems associated with schizophrenia...with mental health.

C3

As a result, to achieve optimal effects, the length and intensity of treatment often needed to be increased, which added a significant financial burden associated with treatment.

##### Patient Engagement in Psychological Interventions

Achieving optimal control of psychotic symptoms with antipsychotics was considered by clinicians an essential part of treatment. They stated that prompt psychotherapeutic interventions were needed if patients’ desire to consume cannabis increased as their psychotic symptoms became less intense. Clinicians believed that peer pressure to use cannabis—combined with patients’ diminished self-awareness related to the effects of cannabis on their mental health—represented a significant challenge for psychological interventions, especially in the more permissive social context related to cannabis use postlegalization in Canada:

Ever since pot was legalized, I’ve noticed a phenomenon for different individuals. It’s that the patients tend to play down how cannabis use can impact their lives.

C3

Certain drug use patterns were a barrier to psychological interventions because arriving intoxicated at clinical visits impeded patient-clinician communication, whereas concurrent drug use (eg, using cannabis to antagonize the effects of *speed*) required a reassessment of psychological intervention targets. Patients viewed engagement in the treatment as an important determinant of achieving optimal results:

...you know, you can also cheat in psychotherapy. You can... I mean there’s... if you’re really determined, you can do it.

P1

Patients’ precarious socioeconomic situations (eg, homelessness) had a negative impact on their engagement in psychological interventions:

Well, it also depends on Maslow’s pyramid...what I mean is that they’re homeless... um...they don’t have any income, what I mean is that, even though you will work on your use...they may not be there yet, you know?

C2

Clinicians were confident that psychological interventions could interrupt the vicious cycle where cannabis misuse contributed to unemployment, low educational attainment, low income, and poor social interactions, all of which could in turn contribute to increased cannabis consumption.

##### Clinicians’ Skills and Experience With Psychological Interventions

Depending on clinicians’ training background, skills, and experience, the type and intensity of face-to-face psychological interventions varied. In clinicians who attended only short-term (1 to 2 days) formal training in psychological interventions, engagement and competency in using psychotherapeutic interventions (eg, CBT, motivational interviewing [MI]) was facilitated by working with highly skilled clinicians in EISs for psychosis. An understanding of and a compassionate attitude toward cannabis consumption habits facilitated a strong therapeutic bond that was viewed as essential in improving cannabis use outcomes. Psychological interventions for CUD were viewed as a component of the comprehensive treatment approach needed by young adults with FEP:

We try to talk about cannabis as well, but we are not there to treat cannabis based on our plans. I think that it’s an integrated approach.

C3

Clinicians used CBT, MI, HR, or psychoeducation as single therapies or in combination and as individual or group therapy, for example, life balance group therapy.

Patients received varying intensity of psychological interventions for CUD, depending on the treatment priorities of the clinician and clinic:

But I don’t do it systematically - because I don’t think about it in a systematic way - and not every time either. But I think that I might do it more than I think...but without even realizing it.

C2

We are, of course, in a clinic, where the priority, at first, is still to make sure that we are treating acute psychotic symptoms.

C10

Clinicians who had experience with treatment protocols for SUDs acknowledged the value of these protocols in providing guidance and facilitating discussions with patients about cannabis consumption. Some barriers to implementing treatment protocols included low patient adherence to treatment and insufficient tailoring of the protocol to individual needs. Good communication within and between intervention teams related to treatment goals was considered of key importance for improving cannabis-related outcomes:

Well, there’s a team (community-based) that does what they want. And then someone else will say, “well, you can use but be careful of this and that.” It makes it so that the teenager never talks about their use issues with their psychiatric team because they’re afraid.

C3

Clinicians frequently lacked the time to deliver well-structured psychotherapeutic interventions. In addition, staff turnover interrupted the continuity of interventions. In clinicians’ opinion, an interdisciplinary approach that included specialists in addiction psychiatry and psychologists could overcome some of these barriers.

#### Behavioral Stage of Change

Patients stated that adequate motivation to change cannabis consumption was a prerequisite for optimal engagement in interventions and achieving cannabis abstinence:

...it could never, even with traditional psychotherapy, it could never do the whole job, and it’s up to you... it’s always like 80/20. You have to put in the majority of the effort and that’s it.

P1

Because for me, they always asked me to stop, stop, stop. But it wasn’t coming from me. So, I don’t stop.

P3

To identify patients’ personal treatment goals, it was important to proactively assess their behavioral stage of change that reflected their motivation to decrease cannabis use:

It’s rarely them that will come and ask for help saying, “hey, I’m here because I have a problem so big that I have to make drastic changes to my life.”

C3

For example, both clinicians and patients explained cannabis abstinence relapse when coercive measures (eg, court order) were implemented by patients’ lack of motivation to change their cannabis use.

Interestingly, clinicians’ and patients’ experience with motivational group therapy shows that patients who are in precontemplation (ie, do not want to change their cannabis consumption) could benefit from low-intensity motivational interventions to help them progress toward more advanced stages of change:

Whereas otherwise, one week, two weeks can go by, and I won’t think about it at all. And then, having to go every week, it’s like... I have more and more now when I smoke outside, I think about it.

P6

Clinicians considered patients who progressed to more advanced stages of behavioral change, including contemplation (ie, ambivalent), preparation, or action stages, as good candidates for higher intensity MI and/or CBT (ie, more frequent and regular sessions). Notably, clinicians agreed that the greatest barrier to psychological interventions was patient unwillingness to decrease cannabis use.

### Strategies for Psychological Interventions

#### Cognitive Behavioral Therapy

CBT was considered an appropriate psychotherapeutic approach for patients with a relatively high functional status (ie, with a full-time job and stable housing) and for those who developed some degree of motivation for changing their cannabis use:

Yes, it’s because they are in precontemplation and when you come up with a CBT approach... it makes um... they look at you... and it’s as if they don’t hear you.

C9

By modifying cognitions (ie, thoughts), clinicians stated that CBT could enable patients to make better decisions about cannabis consumption and to improve their social interactions:

What they enjoy most in life is chilling out. They don’t have any other interests, and my job is to help encourage them to try other things.

C9

This view was echoed by patients who expected psychological interventions to teach them techniques useful for replacing the rewarding effects of cannabis with other activities and for facilitating positive thinking:

I think that we’re just looking for the effect of the substance. That effect would have to be reproduced by something less dangerous or something like that. Because I think if... we're always looking for the effect. It’s the effect of the drug that a user looks for and that’s why they use.

P5

If you are addicted to it, then, of course, you see the negative in everything all the time, so, of course, you want to smoke all the time, and then you will relapse, you know?

P10

Useful behavioral distraction strategies included encouraging patients to discover interests and passions, engaging in activities (eg, talking to friends, watching movies, playing games, or listening to music), and congratulating them on being persistent in their choice. Patients were aware of the importance of engaging in occupations that divert their attention away from consuming cannabis to achieve personal cannabis use goals and better manage cravings:

There’s like playing sports um... like when you want to use, well you watch a TV show or a movie instead. You occupy yourself... you fill your head with other things.

P8

#### MI and HR

Clinicians used MI to encourage patients to examine their thoughts and feelings and increase their awareness of problems triggered by cannabis abuse, for example, anxiety. They considered MI effective in lowering patients’ mental health stigma and increasing their acceptance of and adherence to specialized treatments. Patients stated that psychological interventions helped them conceptualize the effects of cannabis on their mental and functional status, identify personal reasons for reducing cannabis use, and understand their consumption habits:

The more I use, the less creative and focused I become. And I did it a lot when I was making music. So then, I was just like... it’s one more reason to stop.

P3

For me, like you said, playing sports um... because for me it’s... Me too, when I actually quit smoking, because it’s like cyclic. It seems like sometimes I smoke and sometimes I just can’t help myself from smoking.

P9

Although clinicians perceived MI to be less effective in addressing CUD in patients unwilling to change their cannabis use (ie, lack of motivation), they continued with low-intensity MI until the patient became more receptive to change. Typical MI questions were centered around the influence of cannabis on patients’ functional status and increasing their perception of self-efficacy corresponding to their short-term achievements:

...how does it impact your studies, your social relationships, in what way? Do you notice a difference in how you feel when you’re using versus when you’re not using? Are you satisfied with that?

C2

What are your achievements? What were you proud of this week? What did you accomplish?

C2

In patients who achieved abstinence, clinicians avoided questions related to cannabis use and focused instead on long-term objectives (eg, employment or school).

For patients who did not want to decrease their cannabis consumption, some clinicians considered that the right approach is to start with HR interventions (eg, buying cannabis with low tetrahydrocannabinol [THC] content or purchasing from legal sources). HR interventions can help severely functionally impaired patients control their cannabis use, which is essential for securing basic life necessities (eg, housing) and avoiding coercive measures (court orders) that were considered less effective and last-resort measures. Encouraging patients to smoke later in the day and after school or work could increase their satisfaction with life, self-confidence, and self-efficacy related to controlling cannabis use:

And they redefine themselves differently than just as a user, it makes them say, “I’m also a worker, that’s what I do in life.”

C2

The HR approach was acceptable for patients because of its emphasis on controlling cannabis use rather than achieving abstinence:

Well, I think that in life you always have to find a balance. It can’t be all black. It can’t be all white. There’s always a little bit of one in the other, kind of like yin and yang.

P2

#### Psychoeducation

Easy access to information related to available cannabis products was valued by patients:

... I was really looking forward to pot being legalized so that I’d have more information. Since it was legalized, I’ve started smoking pot that’s a lot lower in THC. And I have less... I don’t have as many psychotic symptoms as before.

P9

Clinicians stated that psychoeducation (eg, information about the effects of THC and cannabidiol [CBD]) could act synergistically with HR toward achieving cannabis consumption goals. They reported that providing information about the effects of cannabis on mental and physical health facilitated patient reflections about cannabis-related lived experiences and helped develop their motivation for treatment. Emphasizing the clinical benefits of cannabis abstinence was considered important as it prepared patients to better deal with more permissive social norms related to cannabis use postlegalization:

And also considering that it’s legal now, there’s a trivialization—even before and after cannabis was legalized—that’s very present socially in Quebec. It means that we have to talk about the effects of the substance.

C7

Clinicians stated that psychoeducation delivered in both individual and group therapy could have complementary effects and that information should cover a broad range of health-related aspects associated with cannabis use without assuming that patients have basic knowledge.

#### Stress Management

Patients expected psychological interventions to help them gain control of their cannabis use by acquiring skills (eg, mindfulness meditation) to deal with general life stressors and cannabis use–related stress (eg, cravings):

That would be one of my goals, in psychotherapy, to deal with the stress I would normally take away with ... by smoking a joint.

P3

Clinicians believed that integrating stress management approaches in the long-term treatment plan could help patients reduce the amount of cannabis used and maintain therapeutic gains:

...once there is a reduction, I make sure that it is always anchored in factors of stress and how to manage them - before starting the next step - you know, we take it step by step.

C6

#### Addressing Concomitant Substance Use

Clinicians stated that in their experience, the most prevalent substances used by young adults with psychosis are cannabis and tobacco, followed by alcohol and amphetamines. Clinicians addressed multidrug use differently: some preferred a global approach to addiction (ie, not drug specific), whereas others used drug-specific psychological interventions and prioritized the most problematic substance for the patient:

Of course, we always ask the person: what’s your biggest issue? Because often trying to work on everything... it scares them. If we say “well, you know what, we will work on your addiction in general.” They're going to say, “well, hang on then”... you know? So that's it, you have to work with them.

C8

There was ambivalence among clinicians regarding interventions simultaneously targeting cannabis and tobacco abuse. Motivated by the fragile mental status of some patients, clinicians generally recommended a sequential approach that starts with addressing cannabis misuse; in their opinion, patients could be overwhelmed by simultaneously reflecting about 2 substances and handling distinct objectives related to substance misuse. Despite acknowledging the negative long-term health consequences of tobacco smoking, clinicians did not view tobacco use disorder as a treatment priority because patients often request support for quitting smoking after achieving treatment milestones related to cannabis use and significantly improve functionally.

#### Addressing Psychosocial Determinants and Global Functioning

Clinicians described the unfavorable psychosocial context of many young adults with FEP: disruption of normal life trajectories, difficulties in social interactions, unmet basic life needs, and inability to pursue personal goals. Consequently, clinicians adopted a holistic treatment approach and simultaneously addressed CUD and patients’ poor functional status:

But... because I, I...I just can’t separate quitting from what he does in life.

C2

They felt that a multidisciplinary intervention strategy could help patients slowly regain control of their lives and increase their adherence and confidence in long-term CUD treatment.

### Incentives

#### Promoting Engagement With Peers and Sense of Achievement

With regard to internet-based psychological interventions, patients suggested that an incentive algorithm contingent on participation in the intervention (eg, completing modules, participating in group discussions) could act as a catalyst for patient engagement in treatment. A reward system could help patients get recognition from their peers and satisfy their personal need for achievement:

...because there are people like me, the “completionist”. As soon as “achievements” come into play, I have no choice but to reach them.

P1

#### Contingency Management

Clinicians valued contingency management (CM), as it facilitated patient reflections about cannabis use habits and allowed them to eventually develop motivation to engage in behavioral change. Some clinicians offered rewards contingent on participating in treatment sessions (eg, group therapies) to patients who initially lacked motivation to change their cannabis use. These clinicians reported that some of these patients changed from precontemplation to contemplation:

In our balance group, well we still do it, you know, they’re in precontemplation and don’t have a desire to change, and the worst thing is that they come just to eat pizza... but they’re there for an hour and we talk about use. It makes it so... in the end, they don’t only come for that.

C9

In contrast, for patients who were motivated but had not yet stopped using cannabis, clinicians advocated against using CM:

And then there are some patients that don’t need it. For the type of people that are already self-motivated. I find that giving them money to come and reflect, it puts them into the position of a patient that needs the clinic to function, you know?

C3

In cannabis-abstinent patients, providing grocery coupons contingent on cannabis-free urine samples was considered effective in maintaining abstinence. A major barrier toward including CM in standard services offered in EISs for psychosis is the absence of funding dedicated to such *intervention*; in this context, offering financial incentives from patients’ own budget (applies to a subgroup of vulnerable patients that benefit from budget management at the clinic) contingent on cannabis abstinence was considered a viable alternative to achieve long-lasting treatment benefits because it actively stimulates them to change their cannabis use behavior:

But I think what’s important is that the person is able to satisfy themselves, on their own, so that they can develop that confidence: “OK, I will allow myself to go to the movies” for example, or “go out with friends”, you know, it’s finding the right way to reward themselves.

C2

Notwithstanding its putative efficacy in increasing patient adherence to scheduled psychological intervention visits or maintaining abstinence, clinicians advised against using CM over long periods to enable patients to develop independence in managing their cannabis consumption before they graduate from the 3-year intensive follow-up program offered in EISs for psychosis.

### General Interest in TBPIs

#### Facilitators of and Barriers to TBPIs

Clinicians mentioned that TBPIs could be considered by patients as less formal than face-to-face interventions, facilitate shared decision making around the therapeutic plan, decrease the probability of confrontational situations triggered by patients’ reluctance to disclose their cannabis use patterns, and offer patients more time to reflect on their cannabis use. Clinicians believed that TBPIs would enable patients’ rapid access to support when in urgent need, for example, when craving cannabis. Although using TBPIs could circumvent some patient-clinician communication barriers (eg, commuting time for appointments), clinicians were concerned that in patients with pronounced avoidant behavior, the use of TBPIs could exacerbate their isolation and social anxiety. Clinicians feared that in some patients, TBPIs could weaken the clinician-patient therapeutic bond that was an important determinant of adherence to treatment:

So, I would explain to them that the possibility exists, but again, it doesn’t take away the meetings, the importance of the meetings and that we will continue to put in the work. But that we could use this tool that could help make things easier to access. I would present it to them kind of like that.

C10

Moreover, both clinicians and patients mentioned that TBPIs could more easily lead to *cheating on the treatment plan* when patient engagement was low; therefore, maintaining human contact was viewed as important to ensure treatment success:

Yes, face-to-face human contact may not be a priority - but there has to be some form of human contact and that someone, somewhere, sees progress.

C6

Patients mentioned that their engagement in using TBPIs could be influenced by the level of social support for treatment (eg, family, friends) and subjective norms (ie, opinion of peers that could regard TBPIs as less reliable than face-to-face interventions):

Well, I think they’d take me less seriously than if it was real therapy.

P8

In the context of psychosis, patients’ poor cognitive abilities could impede their ability to use TBPIs and delusions (eg, being spied on via the internet) could decrease their willingness to use TBPIs. Clinicians believed that TBPIs could increase access to psychological interventions in remote areas and in individuals with subthreshold psychosis who misused cannabis and who were not treated in specialized mental health services. Patients described TBPIs as a comfortable and accessible alternative to face-to-face psychological interventions but expressed concerns about costs associated with using their personal data plan.

The acceptability of TBPIs for clinicians was dependent on their readiness to integrate internet-based applications in the traditional model of clinical work, their general skills in using technology, and their training in using new applications. Some clinicians who were familiar with telepsychiatry (eg, for patients with anxiety or depression) saw the value of TBPIs in providing visual interactions with patients and considered them an option for group therapies. Clinicians highlighted that developing an application to address cannabis misuse in young adults with psychosis is timely in the context of uncertainties related to the long-term impact of legalization of cannabis consumption. They mentioned that such an application could increase the intensity of psychological interventions, decrease clinician workload, and help less formally trained clinicians deliver psychological interventions for cannabis misuse in a consistent way. To achieve these goals, clinicians suggested that the application should be tailored to patient treatment goals and be offered to all patients. In addition, clinicians requested adequate training on how to use the application and suggested the presence of a clinician promoter of the application on site. To facilitate its implementation into practice, they would use an informal, nondirective approach in promoting the application, especially in patients that are unmotivated to change their cannabis consumption:

But we can say, listen, there’s this new thing that we can try together if that’s okay with you? You will see, you know, if you like it or not, it's really your choice. This is just one more thing that we’re offering you. You don’t force it on them and explain it more clearly by saying, “I think that it could help.”

C2

Other suggestions to facilitate a successful implementation provided by clinicians included walking the patient through the functionalities of the application, providing assistance as needed, and using a demo version to advertise the application in the waiting room.

#### Technology-Related Attitudes and Beliefs

Clinicians acknowledged the widespread use of technology (eg, smartphones and apps) among young adults for whom it is an integral part of their social life:

And our patients will be less and less... I think teenagers are becoming less and less able to express themselves verbally. And more and more able to do everything using technology - both their social skills and their connections to each other or to others and all that.

C3

Clinicians highlighted that the poor socioeconomic status of some patients explained their lack of familiarity with newest technologies, use of outdated devices, and reliance on free wireless networks for internet access and on free text messaging services to communicate with peers. In their opinion, inconsistent access to technology was sometimes a consequence of pawning their devices to buy substances. Nevertheless, clinicians considered it important to capitalize on the high rate of technology use among young adults and implement TBPIs for young people with psychosis who abuse cannabis. For some patients, barriers to using TBPIs included inadequate protection of confidentiality and personal data over the internet, potential health harms of technology (eg, cell phone radiation), and preference for in-person interactions:

I’ll tell you what, secure technology is impossible.

P10

I’m becoming a bit scared of new technology... I stay away from using it. And I like to sleep far away from my phone.

P3

Um ... it would be if you weren’t talking to a robot. I think that’s the most important thing. It’s that you have human contact ... that there’s like human interaction.

P8

Clinicians also questioned the confidentiality of personal data when using internet-based applications and platforms and ethical implications of using TBPIs in acutely distressed patients.

### Tailoring of TBPIs

#### Outcome Measures of Interest for Clinicians

Frequently, outcomes of interventions for SUDs were defined and prioritized jointly by clinicians and patients and were reassessed on a regular basis. Apart from outcome measures directly related to cannabis use (ie, frequency of use, quantity, abstinence, and relapse), clinicians were interested in patients’ motivation (ie, stage of change), their confidence in achieving and maintaining cannabis reduction goals, and their perception of their own ability to resist the temptation to use. In addition to cannabis, clinicians monitored the type, number, and frequency of use of other substances, for example, alcohol, amphetamines, and tobacco. They regularly assessed patients’ compliance with the recommended daily occupational schedule (eg, eating, working, studying, or sleeping), with the goal of assisting them in achieving personal long-term life objectives, for example, graduating from school or finding a permanent job. Clinicians were also interested in assessing patients’ mental health status, quality of life, and quality of relationships with family and friends. Clinicians preferred that TBPIs collect data about how often it helps patients resist the urge to consume cannabis and the locations or contexts in which the application is accessed (eg, at home or while being bored or stressed) to better understand the triggers of cannabis use. Finally, they were interested in patient satisfaction with the application, frequency of application use, and patient rating of the helpfulness of the application in increasing their reflection on cannabis use and achieving consumption goals.

#### Strategies for Delivering TBPIs

##### Communication and Support

Patients and clinicians agreed that TBPIs must align with the multifaceted support needs of young adults with psychosis and CUD. Often individuals with early psychosis experience a state of social isolation, and their main pillar of support is their therapist. Patients and clinicians emphasized the need to facilitate interactions with family, friends who do not consume cannabis, other patients with psychosis and CUD (eg, group therapies), and community services for people with mental health problems. For patients with poor family support, working in synchrony with intervention workers from community organizations, including partners from supervised housing facilities, shelters, and residential treatment facilities, was considered critical:

PortageTSTM [a drug addiction rehabilitation center], they really do wonders. They’re more into creating therapeutic communities where everyone helps each other, and you can build yourself up as a new person in a community that accepts you - unlike communities on the street that aren’t as healthy - to heal you.

C7

Both patients and clinicians suggested that TBPIs could broaden patients’ support systems by providing information about available community resources and integrating static communication channels (eg, text based, where a significant time lag exists between exchange of ideas) and live communication channels (eg, text, audio, or audio video) that enable real-time exchange of information. Clinicians and patients favored a balanced human-technology psychotherapeutic approach and highlighted that TBPIs should not be limited to static content (eg, therapy modules) or robot-like interactions (eg, automated answers) but also include live interactions to enable immediate support from health professionals and patients’ social circles.

##### Format and Structure of the TBPIs

With regard to the format of TBPIs, patients suggested that static information could be offered in text, audio (for situations when reading is not convenient), or video format (eg, motivational videos). They suggested a regular update of information to maintain a high level of interest in the intervention. Patients would appreciate an interactive application that allows discussions with their clinician and personalization of the content (eg, based on individual answers to questions during the intervention). Divergent opinions were expressed by young adults with regard to accessing TBPI modules, as some were against the idea of having free access to all modules from the beginning and others deferred to individual preference and suggested having the option of selecting the frequency at which the modules would be unlocked. In terms of the TBPI structure, patients preferred to have the option of both individual and group interactions. Some preferred to participate in a TBPI about 2 times per week for approximately 10 minutes per session. Reminders in the form of weekly notifications were considered useful to maintain active participation in the intervention; however, too frequent notifications were considered intrusive by patients.

Clinicians highlighted the importance of tailoring the format, content, and structure of the TBPI to patients’ motivation to change cannabis use, to their cognitive difficulties, and to the presence of psychotic symptoms. They recommended MI, CBT, and psychoeducation modules to be delivered in a simple and friendly language combined with graphics or images to facilitate patients’ reflection of cannabis use. An example of easy-to-understand content included testimonials of young people about the lived experiences of psychosis and CUD. To maintain an adequate intensity of the intervention, completion of at least one module per week (not exceeding 30 min) was recommended. Some clinicians were concerned that a TBPI would not be effective in decreasing cannabis use for patients in the precontemplation stage, independent of the intensity of the intervention. For patients who are ambivalent, clinicians estimated that TBPIs lasting 2 to 4 months would facilitate a clinically significant reduction in cannabis use; if a patient’s objective is abstinence, a longer duration (3-6 months) was recommended. Clinicians emphasized that, at the end of TBPIs, patients should be offered booster modules every 2 to 3 weeks (to follow up on patients’ objectives) for a minimum duration of 3 months to maintain cannabis use therapeutic gains. Throughout the TBPI period, clinicians recommended the use of reminders to stimulate patient reflections about cannabis use and ensure adequate participation in the intervention and optimal adherence to standard of care treatments. In their opinion, patients with control of psychotic symptoms (eg, paranoia) would benefit most from TBPIs. Some clinicians hesitated to provide recommendations for the length of TBPIs and follow-up because of variability in the efficacy of psychological interventions among patients.

#### Application Needs and Preferences

Clinicians agreed with patients that the application hosting the psychological intervention should offer a dedicated space for accessing information about the different types of cannabis (eg, Sativa or Indica), effects of consumption (eg, addiction potential or impact on psychosis), or effects of THC and CBD:

...you know like the type of users, because you know, sometimes the people that use Indica are not going to be the same type of people that are going to use Sativa.

P4

Providing up-to-date information could correct misconceptions and facilitate patients’ reflections about cannabis use. Recording the quantity of cannabis used was of interest to patients, as it could help them monitor associated costs; however, some had reservations about providing this information daily or disclosing it to clinicians. On the other hand, clinicians considered the data recorded by the application to be more reliable than patients’ estimates of cannabis use provided during face-to-face visits. Patients preferred the application to be multifunctional beyond simply hosting the psychological intervention modules:

You know that apps can do a lot now... like Amazon it’s not just for buying stuff. There are so many other things you can do.

P6

Additional functionalities suggested by patients included monitoring physical exercise and budget, providing up-to-date information about activities available in their area, and suggesting stress relief methods (eg, meditation techniques). Clinicians and patients suggested that the application could be helpful in planning daily activities (eg, eating or sleeping) and achieving a balanced lifestyle, including improved control of cannabis use:

But it’s... maybe it has another use, that it makes you more aware, there’s something that you want to do about it. And it goes beyond just stopping smoking and it will improve your quality of life.

P6

#### Technology Needs and Preferences

Aligned with their application needs, patients and clinicians suggested the following technological solutions: internet links to reliable sources of information about cannabis, informational and motivational videos, and add-on applications and widgets (eg, fitness, budget management, logbook for cannabis consumption, network games, daily planner, and scoreboard for reward points accumulated based on progress in the intervention and goals achieved). Patients suggested that customization and personalization features (eg, avatars, questionnaires about personal interests, and hobbies) could increase their interest in using the application. Reminders (eg, to participate in the intervention or to attend scheduled visits) could take the form of alarms or text notifications. The application could have various embedded communication tools, such as simple text messaging, chat, forum, and video sharing. Through the application, patients preferred to have rapid access to contact details of key resources, such as their therapist, mental health and addiction facilities in the community, and public services for nonurgent health issues (ie, Info Santé in Québec). Finally, both categories of participants emphasized that using a design that appeals to young people (eg, colors, images, or interactivity) and ensuring flawless functionality on multiple platforms (eg, iPhone or Android) are necessary to maintain optimal application use and retention in the psychological intervention.

## Discussion

### Principal Findings

Our findings advance the qualitative literature by exploring and comparing the views of patients with early psychosis and clinicians related to the use of psychological intervention to treat CUD and their needs and preferences for TBPIs in the context of EIS for psychosis. Our results showed that the type and intensity of face-to-face psychological interventions for CUD were variable and depended on clinicians’ training background, skills, and experience. Similar to previous studies, we found that structural factors (ie, lack of time and staff turnover) represent barriers to psychological interventions [13,28]. Clinicians viewed TBPIs as a useful addition to their toolbox of interventions and as a way to circumvent some of these barriers and increase the consistency of services offered in FEP clinics.

We found that patients’ motivation to change cannabis use was a central psychological intervention target and an important determinant of the type of psychological intervention used. Clinicians often relied on the Transtheoretical Model [29] as a framework to assess patients’ motivation and jointly decide on treatment goals, select the type of psychological intervention, and monitor treatment efficacy. Similar to the results of a previous survey of Canadian EIS for psychosis [13], we found that patients’ lack of motivation (ie, precontemplation) represented a major barrier to psychological interventions for CUD. Despite clinical evidence showing that brief MI interventions are effective when used in synchrony with HR in individuals with SUD [30], for patients in the precontemplation stage, clinicians preferred HR. In these patients, normal life was disrupted (with serious implications on housing, finances, and social interactions) and improving their functional status was a priority intervention target for clinicians. Clinicians combined HR with psychoeducation (to correct misconceptions and facilitate patients’ reflection about cannabis use) and concentrated more on MI, once patients started building self-motivation. As the theoretical underpinning of both approaches is to engage individuals in discussion to activate motivation for achieving long-term cannabis use–related goals, it may at first sight appear surprising that clinicians used mostly HR alone in patients in precontemplation. Possible explanations include inconsistent use of standardized protocols in EISs for psychosis, heterogeneity in staff psychological intervention training, and other treatment priorities, for example, treating acute psychotic symptoms.

The use of CM was restricted to patients in precontemplation (contingent on attending scheduled group therapy) and maintenance (contingent on providing cannabis-free urine specimens) stages. Clinicians emphasized the importance of developing and maintaining intrinsic motivation and patient autonomy in controlling cannabis use to achieve long-lasting cannabis consumption goals. Therefore, using CM only in conjunction with other psychological interventions (eg, MI, CBT, or psychoeducation) was considered potentially beneficial in achieving long-term reductions in cannabis use. This opinion is empirically supported by recently published data from the Contingency Intervention for Reduction of Cannabis in Early Psychosis randomized controlled trial (RCT), which showed that cannabis use and abstinence rates were not statistically different between the CM and computer-based psychoeducation intervention arms at 3- and 18-month follow-ups [28,31]. Our results suggest that offering nonfinancial incentives as part of the TBPIs could be considered a strategy to increase patient engagement in the intervention, are social reinforcement techniques (eg, certificates for achieving treatment milestones), and are generally appreciated by individuals receiving interventions for SUD [32,33].

We highlighted that social isolation among young adults with psychosis could explain their perceived need for regular communication with clinicians, family, friends, peers with similar lived experiences, and community mental health services. In their study, Fortuna et al [34] described social isolation as a hallmark of people with serious mental health illness, with approximately 60% reporting feeling lonely; the authors identified addressing loneliness as the primary unmet need in these individuals. In our study, patients and clinicians opted for a balanced therapeutic approach that used both technology-based and face-to-face communication. Informed by Roger’s model of diffusion of innovation adapted for eHealth interventions, Eysenbach [35] highlighted that increased personal contact with clinicians, receiving positive feedback from health care professionals, and facilitating peer-to-peer communications could decrease the nonusage attrition rate of TBPIs. Our results align with those of Byrne et al [36], who reviewed qualitative research studies to explore priorities in treatment outcomes for individuals with psychosis and highlighted the need to improve social and functional abilities and satisfaction among these patients. In our study, participants mentioned that smartphones are frequently used to engage in social communications, which aligns with the findings of Schlosser et al [37,38], who used an RCT and a smartphone-based intervention to improve motivation and quality of life in people with recent onset schizophrenia spectrum disorders. In their feasibility study, 75% of participants owned a smartphone and 96% reported using a social media platform [37]. Importantly, Schlosser et al [37,38] used qualitative research methods to elicit participant preferences and tailored the intervention to their most important values: to have a sense of control over their future and deepened relationships with family and friends.

Our synthesis of clinicians’ and patients’ preferences related to the functionalities and design of a TBPI aligned with the results of other studies using qualitative and quantitative methods. In their systematic review of studies using mobile application-based interventions in individuals aged 13 to 26 years with prodromal and FEP, Camacho et al [39] showed that live communication platforms (eg, chat or forum) were widely used and responded to patients’ need for rapid support by enabling the sharing of lived experiences and information. In their survey of young adults with FEP related to their preferences of using technology to deliver specialized psychiatric services conducted in Québec before cannabis legalization, Lal et al [40] found that 91% of young adults with psychosis would like to receive information related to mental health, psychosis, and recovery in general. Results of a population-based survey [2] conducted in Québec postlegalization (2019) showed that approximately 25% of adults aged 18 to 34 years considered regular cannabis consumption to be minimally related to health risks and approximately 75% of the same age group believed that recreational use of cannabis was acceptable. In this context, providing accurate information about the effects of cannabis on mental health could prepare youth with psychosis to better cope with permissive social norms. Consistent with our results, Bucci et al [41] and Schlosser et al [37] highlighted the importance of using a casual, friendly, and nonstigmatizing approach and using an appealing design that could resemble a social media application rather than a clinical tool. The authors highlighted the importance of personalizing the application with individual therapy goals and features of interest (eg, communicating and sharing photos with peers), minimizing repetition of content, and adapting the frequency of reminders (ie, notifications) to their preferences [37,41]. Taken together, these measures could facilitate the assimilation of TBPI into patients’ daily routines and increase their participation in the intervention.

This study had several limitations. First, although we used a purposeful sampling approach to maximize the representation of the diversity of young patients with FEP, we recruited only patients treated at the JAP clinic located in the large metropolitan Montréal area and their opinions could be different from the opinions of patients followed up at clinics located in semiurban or rural areas in Québec or other jurisdictions. Second, because of the predominance of male patients in EISs for psychosis, we recruited more males and the voice of females was underrepresented. Third, we did not capture the perspectives of other persons that play a crucial role in the care of individuals with early psychosis and CUD, such as family members and community mental health workers. Finally, from a reflexive standpoint, we acknowledge the probable influence of our global research aims on data collection and analysis, specifically our goal of developing innovative interventions for the treatment of CUD in young adults with psychosis.

### Conclusions

As it stands, research on smartphone-based psychological interventions for young adults with psychosis is limited but increasing [39]. Research into the effects of internet-based interventions on decreasing cannabis consumption in young adults with psychosis is in its incipient stage [19] but is gaining momentum [42,43], with increasing demand for eHealth interventions and the number of jurisdictions that are legalizing the recreational use of cannabis. This qualitative study fills an important research gap related to patients’ and clinicians’ perceptions of psychological interventions and the use of technology to include these interventions in the clinical toolbox for patients with CUD and FEP. We synthesized and compared patient and clinician views and experiences and provided categories of factors that could guide the development of internet-based psychological interventions tailored to their preferences. Future research using quantitative methods to evaluate patient preference with regard to TBPI for CUD in youth with psychosis is needed to validate our findings.

## Appendix

Multimedia Appendix 1 Focus group topic guide.

Multimedia Appendix 2 Interview guide for clinicians.

## Abbreviations

CBD: cannabidiol
CBT: cognitive behavioral therapy
CHUM: Centre hospitalier de l'Université de Montréal (University of Montreal Health Centre)
CIHR: Canadian Institutes of Health Research
CM: contingency management
CUD: cannabis use disorder
EIS: early intervention service
FEP: first-episode psychosis
HR: harm reduction
JAP: jeune adultes souffrant de psychose (young adults with psychosis)
MET: motivational enhancement therapy
MI: motivational interviewing
RCT: randomized controlled trial
SUD: substance use disorder
TBPI: technology-based psychological intervention
THC: tetrahydrocannabinol
