# Supplementary material for: Technology-Based Psychological Interventions for Young Adults With Early Psychosis and Cannabis Use Disorder: Qualitative Study of Patient and Clinician Perspectives
Source: JMIR Form Res. 2021 Apr 5;5(4):e26562. doi: 10.2196/26562 (PMC8056294; doi:10.2196/26562)
Supplement: Multimedia Appendix 1 [file formative_v5i4e26562_app1.docx]

**Appendix A**

1. **FOCUS GROUP TOPIC GUIDE/QUESTIONS FOR PATIENTS**

Introduction of the focus group and definition of psychological treatment (about 5 minutes):

- **Definition of psychological treatment**. A psychological treatment (psychotherapy) for a mental disorder, for behavioral perturbations, or for any other disorder causing suffering or psychological distress, is a treatment that focusses on promoting significant changes in a person’s cognitive, emotional, or behavioural functioning, interpersonal system, personality, or health.

**TOPICS/QUESTIONS**

1. **Treatment needs (intervention targets)** (about 10 min)

| 1. Besides looking for help from a professional, what do you think **YOU** can do to stop or decrease cannabis consumption? |
| --- |

| 1. In what ways do you think **psychotherapy** could help you to stop or decrease cannabis consumption? What are your needs from psychotherapy? |
| --- |

Definition of technology-assisted psychological treatment and presentation of examples of interventions (about 10 minutes):

- **Definition of technology-assisted psychological treatment.** As opposed to face-to-face psychotherapy–where the treatment is delivered by a therapist–in technology assisted psychotherapy, the treatment is offered by using applications (on a computer or smartphone), text messages, or the phone. In technology-assisted psychotherapies, personal contact with an actual therapist is minimal and specific therapeutic messages for improving psychological well-being are almost exclusively exchanged with the patient through computers and/or phones.

1. **General interest in technology-assisted treatments (interventions)** (about 15 min)

| 1. Sometimes technology-assisted platforms (e.g., web-based psychotherapeutic sessions, text messages, phone calls) are used to assist patients in decreasing cannabis consumption. What are your thoughts about **receiving psychotherapy** with the help of technology-assisted platforms? |
| --- |

| 1. Technology-assisted psychotherapy to decrease or stop cannabis use has been used. Examples include web-based psychotherapeutic sessions, text messages, and phone calls. Please think of **any** technology-assisted psychotherapy option **that would be of interest for you**, including but not limited to the examples provided. |
| --- |

| 1. **What do you like best** about the technology-assisted psychotherapy option(s) of interest to you (that you mentioned before). |
| --- |

1. **Barriers and facilitators related to using technology-assisted treatments (interventions)** (about 15 min)

| 1. What would **discourage** you from using technology-assisted psychotherapy throughout the treatment? Please provide us any thoughts on this subject. |
| --- |

1. **Specific opinions about the implementation of technology-assisted treatments (interventions)** (about 25 min)

| 1. Technology skills vary among users. For the technology-assisted psychotherapy option(s) mentioned before as acceptable for you, **what assistance would you need** to make them easier for you to use? |
| --- |

| 1. Technology-assisted psychotherapy for decreasing cannabis consumption comprise multiple sessions. **How often** (e.g., daily, weekly) **would you prefer to use** technology as part of your treatment plan? |
| --- |

| 1. **What is**, in your opinion, **an acceptable length of time** of a technology-assisted psychotherapy session for decreasing cannabis use? What **factors influence** your preference? |
| --- |

| 1. Let’s think about **attending all recommended** psychotherapy sessions. **Compared to** face-to-face psychotherapy, **what could make you decide to quit** a technology-assisted psychological treatment? |
| --- |

| 1. What would your family/friends think about **YOU** using a technology-assisted psychotherapy for decreasing cannabis use? |
| --- |

1. **Closing questions** (about 15 min)

| 1. Of all the things we discussed, what is the most important to you? |
| --- |

During the focus group, the research assistant will write a summary (5 minutes). Then, the following questions are asked:

| 1. Is this an adequate summary? |
| --- |

| 1. The moderator reviews the purpose of the study and then asks the participants: Do you have anything else to add? |
| --- |
