# Supplementary material for: Technology-Based Psychological Interventions for Young Adults With Early Psychosis and Cannabis Use Disorder: Qualitative Study of Patient and Clinician Perspectives
Source: JMIR Form Res. 2021 Apr 5;5(4):e26562. doi: 10.2196/26562 (PMC8056294; doi:10.2196/26562)
Supplement: Multimedia Appendix 2 [file formative_v5i4e26562_app2.docx]

**Appendix B**

**INTERVIEW GUIDE FOR CLINICIANS**

TOPICS/QUESTIONS

Introduction of the study and the objectives of the interview (about 5 minutes)

1. **Treatment needs (intervention targets)** (about 10 minutes)

| 1. In patients with psychosis and cannabis use disorders (CUD), what **intervention(s)** do you think is/are most effective for decreasing cannabis use? Why do you think this/these **intervention(s)** is/are effective? |
| --- |

| 1. What **psychotherapies** would you recommend to decrease cannabis use in patients with psychosis and cannabis use disorders (CUD)? Why would you recommend these **psychotherapies**? Examples of psychotherapeutic approaches include cognitive behavioral therapy (CBT) and motivational interviewing (MI). |
| --- |

1. **General interest in technology- assisted treatments (interventions)** (about 15 minutes)

| 1. What is your opinion related to **using technology-assisted platforms** to deliver psychotherapeutic interventions (e.g., CBT, MI) for decreasing cannabis use in patients with psychosis and CUD? Examples of technology-assisted platforms are web-based, computer-based, and text messages. |
| --- |

| 1. In your opinion, **what technology-assisted platform is best suited** to deliver psychotherapeutic interventions in patients with psychosis and CUD, and why do you prefer this platform? |
| --- |

| 1. How do you think **patients** with CUD and psychosis would **accept the idea of using** a technology-assisted psychotherapeutic intervention? Why do you expect this reaction? |
| --- |

1. **Intervention-specific topics** (about 35 minutes)

| 1. To assess the efficacy of a technology-assisted psychotherapeutic intervention to decrease CUD, what **outcomes** would be of interest for you? Examples of frequently used outcomes are frequency and quantity of cannabis used, dependency, withdrawal, craving, etc. |
| --- |

| 1. When using **technology-assisted** psychotherapeutic interventions, compared to in-person delivered psychotherapy, what are **possible barriers** to achieving a reduction of cannabis use in patients with psychosis and CUD? |
| --- |

| 1. Compared to in-person delivered psychotherapeutic interventions, how would a technology-assisted psychotherapeutic intervention **influence patient adherence to the treatment**? |
| --- |

| 1. How could the barriers you suggested previously be **effectively addressed** in order to achieve an optimal decrease of cannabis use? |
| --- |

| 1. What is your recommendation related to the **total** **length (i.e., weeks)** of the psychotherapeutic intervention to achieve a **clinically significant reduction** in cannabis use? |
| --- |

| 1. What **intervention components** would you recommend to ensure a **long-lasting** effect (i.e., over 6 months) of the intervention on cannabis consumption? |
| --- |

| 1. What is your opinion about using **contingency management** as part of the psychotherapeutic intervention? |
| --- |

| 1. What are your recommendations for a technology-assisted psychotherapy in patients with psychosis and CUD who are tobacco smokers? (E.g., do they need more intensive interventions, would you assess if the intervention had an effect on tobacco smoking?) |
| --- |

1. **Closing questions** (about 5 minutes)

| 1. Suppose that you had one minute to summarize the opportunity to integrate technology-assisted psychotherapy in the treatment plan for CUD. What would you say? |
| --- |
